# Level of Young People Sexual and Reproductive Health Service Utilization and Its Associated Factors among Young People in Awabel District, Northwest Ethiopia

**DOI:** 10.1371/journal.pone.0151613

**Published:** 2016-03-18

**Authors:** Atitegeb Ayehu, Teketo Kassaw, Getachew Hailu

**Affiliations:** 1 Students Clinic, Debre Markos University, Debre Markos, Ethiopia; 2 Department of Public Health, College of Medicine and Health Sciences, Debre Markos University, Debre Markos, Ethiopia; National Institute of Health, ITALY

## Abstract

**Background:**

Currently in Ethiopia, young people’s sexual and reproductive health services are limited and there is a growing issue of confidentiality and affordability of these health services. Moreover, the available services provided are not sensitive to the special needs of young people. Therefore, this study was aimed to assess young people’s sexual and reproductive health service utilization and its associated factors in Awabel district, Northwest Ethiopia.

**Methods:**

A community based cross-sectional study was conducted among 781 randomly selected young people using a pre-tested structured questionnaires in Awabel district, Northwest Ethiopia. Data were entered into Epi data version 3.1 and analyzed using SPSS version 16.0 software.

**Results:**

The mean age of respondents were 17.80 (+ 2.65) years. About 41% of young people had utilized sexual and reproductive health services. Young people from families of higher family expenditure, lived with mothers, participated in peer education and lived near to a Health Center were more likely to utilize sexual and reproductive health services. Furthermore, those who had a parental discussion on sexual and reproductive health (AOR (95% C.I): 2.23 (1.43, 3.46)) and ever had sexual intercourse (AOR (95% C.I): 1.88 (1.30, 2.71)) were more likely to utilize the service than their counterparts. On the other hand, those young people lived with their father and had a primary level of educational attainment was less likely to utilize the service.

**Conclusion:**

Utilization of sexual and reproductive health services is low which needs a great attention where; if not intervened, young people might engage in risky sexual activities. Therefore, it needs a concerted effort from all the concerned bodies to improve their service utilization and thereby reduce the burden of young people’s disease and disabilities associated with sexual and reproductive health.

## Background

The world population is composed of 18% of adolescents (10–19 years) and 26% of young people (10–24 years) [1]. Young people, aged 10 to 24 years, in Ethiopia constitute the largest population proportion; over 21 million young people, i.e., approximately 22% of the country’s total population [2,3]. Young people from sub-Saharan Africa countries are more at risk of sexual and reproductive health problems than those young people from around the world [4]. Youths from this region contribute the highest risk of sexually transmitted infections; for instance, over half of all new HIV infections are among them [4].

It is evident that investing in the health of young people is vital for any country’s socioeconomic development [5]. Adolescents’ access and utilization of sexual and reproductive services is limited due to sociocultural norms and taboos (leading them to fear and feel shame), judgmental attitude of service providers, lack of confidentiality and privacy, costs and lack of SRH knowledge, and an unfavorable attitude of parents and negative community perceptions towards health seeking behaviors of adolescents [6]. Some health care providers had an unsupportive attitude towards providing sexual and reproductive health services to unmarried adolescents; even about 13% of them stressed that there should be penal rules and regulations against adolescents who practiced pre-marital sexual intercourse [7].

Barriers to utilizing and accessing sexual and reproductive health (SRH) services for young people are continuing today. Most of the reasons why young people haven’t utilized SRH services were distant health facilities, poor quality of service, inconvenient service locations, inconvenient hours of operation, unaffordable service costs, they didn’t know where to go and feeling healthy [8]. They didn’t have the basic sexual and reproductive health knowledge and didn’t have access to affordable and confidential SRH services. Most youth didn’t feel comfortable in discussing sexual and reproductive health matters with their family members rather seek information from friends and other sources [9]. As compared to adults, youths were less informed, less experienced and less comfortable in accessing and utilizing sexual and reproductive SRH services [9–11].

A study done in Bahir Dar, Ethiopia indicated that the barriers in utilizing reproductive health services were inconvenient health facility operating hours and fear of being seen by parents or people whom they know [12]. Similarly, parental disapproval, lack of basic information and pressure from partners deterred them in accessing and utilizing the services [13]. A higher age group and experience of reproductive health problems were positively associated with utilization of youth reproductive health services, but it was lower among those who cannot afford to pay for the services they received [12]. Utilization of youth reproductive health services was significantly associated and higher among those whose age was 15–19 years, had reproductive knowledge, ever discussed on reproductive health issues and had the secondary level of educational attainment [13].

A study done in Gondar town, Ethiopia showed that utilization of family planning services was significantly associated and higher among those adolescents who had secondary level of educational attainment, had discussion with family/relatives, peer groups, sexual partners and teachers, had a romantic sexual relationship and had a longer (greater six months) sexual relationship were positively associated with family planning service utilization [14]. Furthermore, utilization of VCT service was significantly higher among females, who had discussion with parents, peers and health workers, ever had sexual intercourse and perception of a risk of HIV/AIDS [14].

Determining the level and assessing factors affecting utilization of sexual and reproductive health services in this young population is very important to improve young people’s sexual and reproductive health service utilization and thereby reduce the burden of young people’s disease and disabilities associated with sexual and reproductive health. Therefore, this study was aimed to assess young people’s sexual and reproductive health service utilization and its associated factors in Awabel district, Northwest Ethiopia.

## Methods

### Study Area

The study was conducted from September 1, 2014 to June 30, 2015 in Awabel district, Northwest Ethiopia. Awabel district is one of the 18 districts in East Gojam Zone, located at a distance of 259 km to the Northwest of Addis Ababa in Amhara Regional State of Ethiopia. The district has a total population of 138687 where 64506 of them are young people and a total of 32253 households [3]. The district has a total of 37 health facilities; six health centers, 28 health posts and three private clinics; and a total of 108 health workers and 56 health extension workers [15].

### Study Design

A community based cross-sectional study was conducted among young people in Awabel District, Northwest Ethiopia.

### Sampling

The sample size was calculated using single population proportion formula based on the following assumptions: the proportion (p) of youth friendly reproductive health service utilization in Harar at 63.8% [8], a 95% confidence level, and margin of error of 5. Using a design effect of 2 and 10% of non-response rate, the final sample size became 781.

The study participants were selected using a multistage sampling technique: out of the 29 kebeles (the lowest administrative unit in Ethiopia) in the district eight kebeles were selected at stage one; one urban and seven rural kebeles. At stage two, 9216 households in each selected kebeles having young people were selected using simple random sampling technique. Finally all the 781 young people (with an age range of 10 to 24 years) were selected using a simple random sampling method with proportionate allocation to size using the Health Post Household Family Folder in each kebeles (sampling frame). Per each household, one young person was participated in the study and two visits were made for absences in the first visits.

### Data Collection

An interview-administered pre-tested close ended Amharic (the local language) questionnaire, which was first prepared in English was used. To avoid information contamination the pretest was done on 40 young people who were residing in one of the unselected kebele of the district and its finding was used to modify the instrument. The questionnaire was mainly focused on socio-demographic characteristics, sexual behaviour, sexual and reproductive health discussion, and sexual and reproductive health service utilization. Young people were briefed about the purpose of the study and data were collected after a verbal informed consent. The data collection process was facilitated by trained ten data collectors with health background and two BSc public health supervisors. The data were collected in the quietest corner of young people’s house where there was no noise and disturbance. The data collection process had taken an average of 40 minutes.

Data quality was assured through careful design of the questionnaire. Data collectors and supervisors were trained in one day about the purpose of the study, the questionnaire in detail, the data collection procedure, the data collection setting and the rights of study participants. Pre-test was done prior to the actual data collection. The collected data were checked for completeness and consistency after each day of data collection by holding a meeting with the data collectors.

### Data Analysis

Data were entered using Epi Data version 3.1 and then exported to SPSS version 21.0 for analysis. Descriptive statistics was used to describe the study population in relation to relevant variables. Bivariate and multivariable models were run to assess any relationship between each independent variables (sociodemographic characteristics, health service variables and sexual and reproductive health characteristics) and outcome variable (young people sexual and reproductive health service utilization, i.e., at least once SRH service used). Crude and adjusted odds ratios were used to ascertain any associations between the dependent and independent variables while significance was determined using 95% confidence intervals. Independent variables found to be significant with p-value less than 0.05 at the bivariate level were included in a multivariable logistic regression model for the dependent variable to control potential confounding variables.

### Ethical Consideration

Ethical approval was obtained from Debre Markos University, College of Medicine and Health Sciences, Research Ethics Committee and a letter of permission was obtained from the Awabel District Health Office. The purpose of the study was explained to young people and a verbal informed consent was obtained from the participants. For those study participants who were under the age of consent, informed written assent was obtained from their parents. Confidentiality of information was maintained by omitting any personal identifier from the questionnaires. The data were stored in a safe place where no one except the principal investigators has access.

## Results

### Socio-demographic characteristics of study participants

Out of 781 randomly selected young people, 746 were participated obtaining a response rate of 95.5%. Above half, 389 (52.1%), of the respondents were females and 545 (73.1%) were rural residents. The mean age of them was 17.80 (±2.65) years and the majority of them, 438 (58.7%) were in the age group of 15–19 years. Nearly one third, 240 (32.2%), of young people had attained a preparatory level of education and 568 (76.1%) were students in occupation. Concerning marital status, 97 (13.0%) were married and most, 471 (63.1%) of them were living with both parents. Nearly one third, 238 (31.9%) of their family had a monthly expenditure of less than or equal to 1000 Ethiopian Birr (Table 1).

**Table 1:** Socio-demographic characteristics of Young People in Northwest Ethiopia, 2015.

Characteristics of respondents (n = 746)		Numbers	Percent
**Age Group in Years**	10–14	107	14.3
	15–19	438	58.7
	20–24	201	26.9
**Religion**	Orthodox	669	89.7
	Muslim	65	8.7
	Protestant	12	1.6
**Residence**	Rural	545	73.1
	Urban	201	26.9
**Educational Status**	Illiterate	50	6.7
	Read and Write Only	39	5.2
	Primary	204	27.3
	Secondary	213	28.6
	Preparatory	240	32.2
**Marital Status**	Single	649	87.0
	Married	97	13.0
**Ethnicity**	Amhara	739	99.1
	Oromo	7	0.9
**Live With**	Both parents	471	63.1
	Father only	112	15.0
	Mother only	66	8.8
	Spouse	97	13.0
**Mother’s Education**	Illiterate	611	81.9
	Read & Write Only	75	10.1
	Primary	57	7.6
	Secondary and Above	3	0.4
**Father’s Education**	Illiterate	278	37.3
	Read & Write Only	375	50.3
	Primary	59	7.9
	Secondary and Above	34	4.6
**Respondents Occupation**	Housewife	74	9.9
	Daily Laborer	100	13.4
	Student	572	76.7
**Mother’s Occupation**	Housewife	717	96.1
	Petty trader	29	3.9
**Father’s Occupation**	Farmer	655	87.8
	Petty trader	48	6.4
	Employed	43	5.8
**Monthly Expenditure (Ethiopian Birr)**	< = 1000	238	31.9
	1001–2000	215	31.5
	2001–3000	129	17.3
	>3001	144	19.3

### Sexual Behavior of Young People

**Fig 1:**
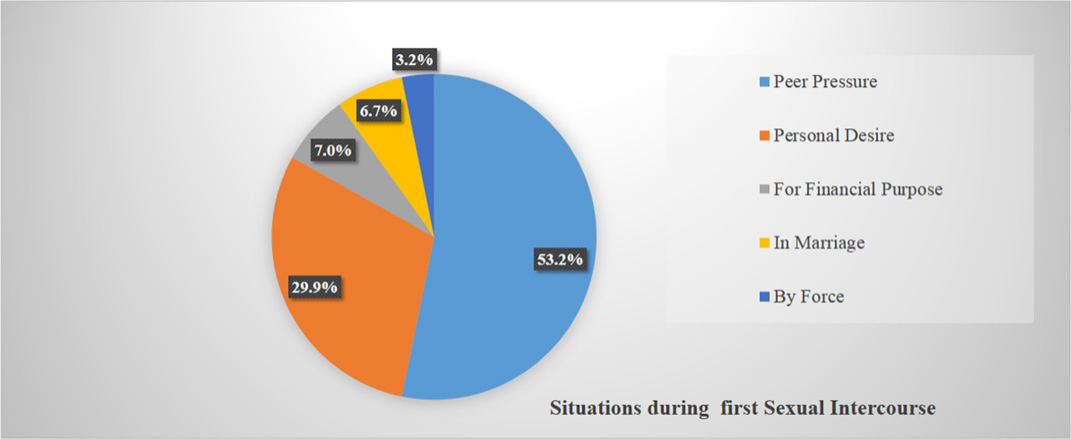
Situations how first sexual intercourse was started among young people in Northwest Ethiopia, 2015 (n = 474).

Of the total 746 participants, 474 (63.5%) of them had started sexual intercourse and for most of them, 395 (83.3%) it was premarital sex. The mean age of first sexual intercourse was at 14.3 with a standard deviation of 2.0 years. During their first sexual intercourse, only 42 (8.9%) of them had used contraception and their major reason to engage in premarital sex was due to peer pressure, 252 (53.2%) and personal desire, 142 (30.0%) (Fig 1). In the past six months; out of them who had a history of sexual intercourse (474), 186 (39.2%) of them were sexually active.

### Utilization of Young People Sexual and Reproductive Health (SRH) Services

Out of the total 746 respondents, 189 (25.3%) had a parental discussion on sexual and reproductive health issues and most of the discussions were made among their mothers and sisters. Two hundred (26.8%) of the young peoples had participated in peer to peer education in their school or village. In the study area, the perceived SRH problems that young people encounter was unintended pregnancy, 429 (57.5%), STI/HIV/AIDS, 416 (55.8%) and abortion, 322 (43.2%).

**Fig 2:**
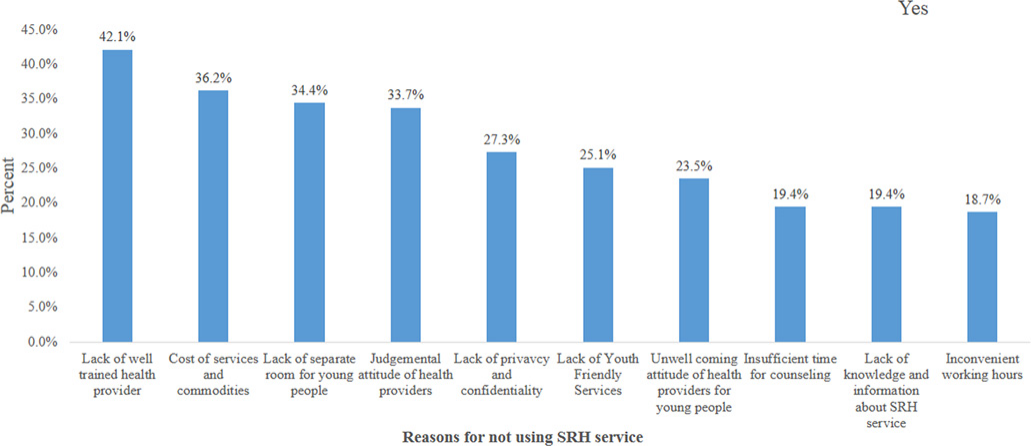
Reasons of young people for not utilizing SRH services in Northwest Ethiopia, 2015 (n = 439).

In this study, in the past six months, only 307 (41.2%) of study participants had utilized sexual and reproductive health services; nearly half, 149 (48.5%), of them had visited Health Centers. The SRH services they had received were SRH information, education and counseling, 157 (51.1%), contraception and / or condom, 78 (25.4%), treated for STI, 53 (17.3%), VCT service, 32 (10.4%) and abortion and post abortion care, 8 (2.6%). Above half, 161 (52.4%), of them were not satisfied with the service they got in those health facilities. The main reasons, i.e., for those who didn’t utilized SRH service (439) were lack of trained health provider, 185 (42.1%), cost of services and commodities, 159 (36.2%), lack of separate rooms for young people, 151 (34.4%) and judgmental attitude of health providers, 148 (33.7%) (Fig 2).

Two hundred twenty five (73.3%) of young peoples had missed the SRH services they need during their health facility visit in the past six months. The main reasons for missing the service were too long waiting time, 96 (42.7%), lack of money for the service, 67 (29.8%) and feeling ashamed when they get their neighbors at the health facility, 34 (15.1%).

With regard to young peoples’ choice of health facility for SRH services, 407 (41.2%) of them wanted to get the service in Health Centers (where the services are provided in a separate room) and for 155 (20.8%) of them it was Youth Centers. Furthermore, their convenient time to get these services was during special hours when there is no more patients, 253 (33.9%) and twenty four hours a day, 203 (27.2%). The preferred SRH service provider for the 162 (21.7%) young people was any provider, whereas for 161 (21.6%) and 160 (21.4%) of them were young provider of the same sex and young provider of any sex respectively.

### Factors associated with use of Sexual and Reproductive Health Service

According to the multivariable analysis young people who were living with mothers were 2.70 more likely to utilize the SRH service while those who lived with fathers were 51% less likely to utilize SRH service when compared to their counterparts (AOR (95% C.I): 2.70 (1.26, 5.78), and 0.49 (0.30, 0.81)) respectively. Those respondents who had attained primary level of education were 74% less likely to utilize reproductive health services than their counterparts (AOR (95% C.I):0.26 (0.12, 0.53)).

Young people of a family having household monthly expenditure of greater than or equal to 3001 Ethiopian Birr were 3.07 times more likely to use SRH services than those with monthly expenditure than their counterparts (AOR (95% C.I): 3.07 (1.82, 5.19)). Respondents had a parental discussion on SRH issues were 2.23 times more likely to utilize SRH services than those who didn’t discuss on SRH issues (AOR (95% C.I): 2.23 (1.43, 3.46)).

Concerning their sexual behaviour, those respondents who ever had sexual intercourse were 1.88 times more likely to utilize the service than those who were not sexually active (AOR (95% C.I): 1.88 (1.30, 2.71)). Young people who had participated in peer to peer education were 1.95 times more likely to utilize the service than their counterparts (AOR (95% C.I): 1.95 (1.29, 2.94)). Similarly, those young people where their nearby health facility was Health Center were 2.36 times more likely to utilize the service than those who were living near Health Post and Private Clinic (AOR (95% C.I):2.36 (1.61, 3.45)) (Table 2). However, residence was a confounding variable on young peoples’ sexual and reproductive health service utilization.

**Table 2:** Factors associated with SRH service utilization among young people in Northwest Ethiopia, 2015.

Variables (n = 746)		SRH Utilization		Crude Odds Ratio (95% C.I.)	Adjusted Odds Ratio (95% C.I.)
		No	Yes		
		n (%)	n (%)		
**Residence**	Rural	307 (56.3)	238 (43.7)	1.00	1.00
	Urban	132 (65.7)	69 (34.3)	1.48 (1.06, 2.08)***	1.33 (0.86, 2.04)
**Live With**	Both Parents	284 (60.3)	187 (39.7)	1.00	1.00
	Father only	45 (40.2)	67 (59.8)	0.44 (0.29, 0.67)***	0.49 (0.30, 0.81)**
	Mother only	54 (81.8)	12 (18.2)	2.96 (1.54, 5.69)**	2.70 (1.26, 5.78)*
	Spouse	56 (57.7)	41 (42.3)	0.90 (0.58, 1.40)	0.63 (0.37, 1.09)
**Educational Status**	Illiterate	27 (54.0)	23 (46.0)	1.00	1.00
	Read and Write	21 (53.8)	18 (46.2)	0.99 (0.43, 2.30)	1.02 (0.40, 2.60)
	Primary	55 (27.0)	149 (73.0)	**0.31 (0.17, 0.59)** ***	0.26 (0.12, 0.53)***
	Secondary	158 (74.2)	55 (25.8)	2.45 (1.30, 4.62)**	1.73 (0.84, 3.53)
	Preparatory	178 (74.2)	62 (25.8)	2.45 (1.31, 4.58)**	2.01 (1.00, 4.06)
**Monthly Expenditure**	< = 1000	118 (49.6)	120 (50.4)	1.00	1.00
	1001–2000	128 (54.5)	107 (45.5)	1.22 (0.85, 1.75)	1.28 (0.82, 2.00)
	2001–3000	88 (68.2)	44 (31.8)	2.18 (1.39, 3.42)**	1.55 (0.92, 2.61)
	> = 3001	105 (72.9)	39 (27.1)	2.74 (1.75, 4.28)***	3.07 (1.82, 5.19)***
**Parental SRH Discussion**	No	290 (52.1)	267 (47.9)	1.00	1.00
	Yes	149 (78.8)	40 (21.2)	3.43 (2.33, 5.05)***	2.23 (1.43, 3.46)***
**Ever had Sexual Intercourse**	No	129 (47.4)	143 (52.6)	1.00	1.00
	Yes	310 (65.4)	164 (34.6)	2.10 (1.55, 2.84)***	1.88 (1.30, 2.71)**
**Participated in Peer Education**	No	296 (54.2)	250 (45.8)	1.00	1.00
	Yes	143 (71.5)	57 (28.5)	2.12 (1.49, 3.01)***	1.95 (1.29, 2.94)**
**Nearby Health Facility**	Health Post	170 (46.3)	197 (53.7)	1.00	1.00
	Health Center	231 (73.3)	84 (26.7)	3.19 (2.31, 4.40)***	2.36 (1.61, 3.45)***
	Private Clinic	38 (59.4)	26 (40.6)	1.69 (0.99, 2.91)	1.02 (0.54, 1.93)

Significant at

*p-value < 0.05

**p-value < 0.01, and

***p-value < 0.001.

## Discussion

This study showed that less than half of the young people in Awabel district had utilized sexual and reproductive health services. Utilization of sexual and reproductive health service was low, especially among young people who live with their father, and had a primary level of educational attainment.

This study revealed that only 41.2% of young people had utilized SRH services available in the nearby health facilities in the past six months. This finding is higher than the previous study done in Machakle district [13] and this difference might be due to difference in time and/or to sociocultural variations. However, this finding was much lower than studies done in Harar [8], Botswana [16] and England [17]. This might be due to differences in the availability or accessibility of Youth Friendly Health facilities or Youth Centers, educational status/level, socio-economic status, urban-rural residence, transportation and cultural variations. Furthermore, lack of information and do not perceive they need the services might have influence in utilizing the service.

Those young people who had a parental discussion on sexual and reproductive health issues were more likely to utilize sexual and reproductive health services than those who didn’t discuss. This was supported by a study done in Gondar town [14]. This might be due to as they freely discussed with their parents, they would have a better knowledge and awareness about SRH services and thus would motivate them to use the service. A study done in Mekele town indicated that female students who had a discussion on sexual and reproductive health issues with family and peers has a positive effect on contraceptive awareness [18].

Young people who had attained primary level of education were less likely to utilize sexual and reproductive health services and it was supported by another two studies done in the Machakle district [13]. Young people who had lower levels of educational attainment might be less likely to convince their parents to have money for SRH services or they might be less likely to discuss about these issues and even might not have a better knowledge about the importance and need of these services.

Having a higher monthly household expenditure was significantly associated and higher for SRH service utilization than their counterparts. A study done in Burkina Faso, Ghana, Malawi and Uganda revealed that financial costs are the common barriers of utilizing SRH services [19]. This monthly household expenditure might include health service expenses and therefore young people of these families might not have financial constraint for sexual and reproductive health services.

Utilization of SRH service was significantly associated and higher among young people who were living with their mother. On the other hand it was significantly lower for those who were living with their father. Those young people who live with their fathers might not discuss openly about SRH issues and/or their need and thus they might be less likely to utilize the service as compared to their mothers. In contrast to this study, a study done in the Machakle district [13] and Gondar town [14] showed that young people who were living with both parents, grandparents and relatives were more likely to utilize than those who were living with single parents. The possible explanation for this would be the more freedom to communicate about SRH issues with relatives than biological family and it might be attributed due to the educational attainment of their parents and relatives.

Young people’s sexual and reproductive health services utilization was significantly associated and higher among those who had participated in peer education on SRH issues and ever had sexual intercourse. This finding was supported by other studies done in Gondar town [14], Kenya [20] and Myanmar [21] as adolescents preferred peer educators as a source of sexual and reproductive information since they considered them knowledgeable and trustworthy [22]. As young people engaged in SRH peer education; they would have a better understanding and their need for the service might increase too. In addition to this, those young people who were sexually active might need the service from being exposed to risky sexual activities as they might need information and counseling service on how to avoid this risky behavior.

Young people where their nearby health facilities were Health Centers were more likely to utilize sexual and reproductive health services than those young people where their nearby health facilities were Health Posts and Private Clinics. Adolescents preferred governmental health facilities for sexual and reproductive health service due to the positive perception of confidentiality, accessibility and cost [19]. This could be due to the privacy, confidentiality, access to trained health provider, availability and cost of services in the nearby health facilities.

### Strength and Limitations of the study

Being a community based study done among urban and rural young people and explored the different independent variables made it strong. However, due to its cross-sectional nature of the study, it is difficult to establish causal relationship between the dependent and independent variables. In addition, it was not a mixed method study where the qualitative study will try to explore in-depth reasons why young people didn’t utilize the service.

## Conclusion

The results of this study showed that utilization of sexual and reproductive health services was below fifty percent. Young people who were living with their mother only, from a higher family monthly expenditure, had a parental discussion on sexual and reproductive health issues, ever had sexual intercourse, participated in peer to peer education and lived near to a Health Center were more likely to utilize SRH services. On the other hand, young people who had a primary level of educational attainment and lived with their fathers were less likely to utilize the health service.

Therefore, there should be a concerted effort; health facilities, health care providers, communities, families and the peers, in accessing and utilizing the service. The tradition of open discussion on sexual matters should be developed at the individual and/ or peers, family and community level, which later improves service utilization and thereby reduce the burden of young people’s disease and disabilities associated with sexual and reproductive health.
